# Process Parameter Effects on Biocompatible Thermoplastic Sheets Produced by Incremental Forming

**DOI:** 10.3390/ma11081377

**Published:** 2018-08-08

**Authors:** Marc Sabater, M. Luisa Garcia-Romeu, Marina Vives-Mestres, Ines Ferrer, Isabel Bagudanch

**Affiliations:** 1 Department of Mechanical Engineering & Industrial Construction, University of Girona, 17071 Girona, Spain; marc.sabater@udg.edu (M.S.); ines.iferrer@udg.edu (I.F.); isabel.bagudanch@udg.edu (I.B.); 2 Department of Computer Science, Applied Mathematics & Statistics, University of Girona, 17003 Girona, Spain; marina.vives@udg.edu

**Keywords:** single, point, incremental, forming, thermoplastics, biocompatible, temperature, process, parameters

## Abstract

There has been increasing interest in the processes that enable part customization and small-batch production in recent years. The prosthetic sector, in which biocompatible materials are used, is one of the areas that requires these types of processes; Incremental Sheet Forming (ISF) technology can meet these requirements. However, the biocompatible thermoplastic polymers formed by this technology have not yet been tested. Hence, the aim of this paper is to cover this gap in our knowledge by analyzing the effects of process parameters on the ISF process with the aim of optimizing these parameters before the actual production of, in this case, customized prostheses. Tests with polycaprolactone (PCL) and ultra-high molecular weight polyethylene (UHMWPE) were performed. Maximum force, surface roughness and maximum depth were statistically analyzed by means of response surface methodology and survival analysis. Spindle speed and tool diameter were shown to be the most influential process parameters in terms of maximum forming force and surface roughness for both materials. In contrast, survival analysis applied to maximum depth showed a greater influence of tool diameter in PCL sheets and a greater influence of spindle speed in the case of UHMWPE.

## 1. Introduction

The paradigms of manufacturing have evolved from craft production to mass production and then from mass customization to what S. J. Hu [1] calls personalization or personalized production. There are several important concepts and technologies that have facilitated the development of personalized production, including open-architecture products, personalization design, on-demand manufacturing systems and cyber-physical systems.

In the context of on-demand manufacturing systems, Incremental Sheet Forming (ISF) emerged to meet the demand for rapid prototyping and small-batch production. The process consists of a sheet being formed by means of a round-tipped tool (or punch), which makes a series of small incremental deformations in the sheet on a predefined path that is governed by a numerical control. It is a simple process that can be applied in a number of different fields, ranging from the automotive and aeronautic sectors to architecture. However, it is struggling to find a place in industrial production beyond the various research efforts carried out in recent years. More recently, the biotechnology sector has been attracting most of these research efforts, which have involved a variety of raw materials (metals, such as titanium or polymers, such as polycaprolactone) as well as different variants of the ISF process (such as Single Point Incremental Forming, or SPIF, i.e., without the presence of a die or Two Point Incremental Forming, TPIF, which fully or partially employs a die).

Within the biotechnology sector, the manufacture of prostheses using ISF process involves two of the key elements of personalized production: design personalization, since customized geometry required is for each patient and on-demand manufacturing systems, since ISF technology makes small-batch production possible. The ISF process also offers certain flexibility since it does not require dedicated machinery that would entail heavy investment. In fact, an ISF production system can be adapted from one of the most common machines found in the workshops: a computer numerically controlled (CNC) milling machine fitted with a clamping system for the sheet. In addition, if a TPIF system is required, the die can be made of wood or resin. Hence, transforming a milling machine into an ISF system is a very affordable solution.

Initially, the raw materials being formed in ISF systems were metallic, such as aluminum alloys (AA1050 or AA3003, for example) and some steels (DC04 and AISI 304, among others). Such materials were widely used because of their good formability. Other metallic materials, such as magnesium alloys and titanium alloys [2] need to be heated to be formed incrementally, resulting in increased complexity and cost. Recently, however, some researchers have focused on polymeric materials and thermoplastics [3], because these have the advantage of being able to be formed at room temperature using the ISF process. 

With regard to the process itself, a common concern for researchers from the traditional literature review for metallic and polymer materials is how the process parameters (step down, sheet thickness, tool diameter and wall angle, among others) affect the finished product; that is to say, how they affect the various specific response variables. The aim of such work is to establish the optimal combination of process parameters for achieving the desired effect on these different response variables. Three of the response variables that have garnered most attention in ISF studies are: (i) the maximum axial force (FZ max), to ensure that the maximum capacity is not exceeded, especially when a machining center has been adapted; (ii) the attained final roughness (Ra), which serves as an indicator of the quality of the finished product; and (iii) the maximum depth (of penetration) (Z) of the tool, which serves as an indicator of the material’s maximum formability before any tearing, wrinkling or breakage occurs. 

Aerens et al. [4], working with aluminum and steel alloys, estimated the steady state axial forming force (FZs) by means of an analytical model that accounts for tensile strength, initial sheet thickness, tool diameter, scallop height, and initial wall angle. The works of Li et al. [5,6] investigated further by seeking an efficient model for tangential force prediction whereas Bahloul et al. [7] focused on minimizing the sheet thinning rate and the tool (or punch) loads. Recently, Centeno et al. [8] shed light on the importance of spindle speed in considering the variation force for metallic materials—a factor which is even more important for polymeric materials [9,10,11]. 

Final roughness is another aspect that has captured attention. Recently, Liu et al. [12] aimed to provide a predictive model and the optimal process parameters for minimizing surface roughness in a study in which they incrementally formed a sheet of AA 7075 O-temper aluminum and investigated four process parameters: step down, feed rate, sheet thickness, and tool diameter. Radu and Cristea [13] discarded sheet thickness and introduced spindle speed for three materials: DC01 steel, 304 stainless steel and AA1050 aluminum alloy. Echrif and Hrairi [14] carried out a similar study on an AA1050-O aluminum alloy sheet.

Formability analysis, regardless of the material, is mainly focused on obtaining forming limit diagrams, e.g., Silva et al. [3], where dedicated equipment is required. However, since the initial stages of the development of ISF technology, alternative and simple formability indicators have been used, such as maximum reachable wall angle and its corresponding maximum reachable depth [2,15].

It is well known that using trial and error methodology for determining the best combination of process parameters in manufacturing processes is expensive and time consuming. Having reviewed the current status of ISF, we have noted that before addressing optimization, a Box-Behnken design (BBD) of response surface methodology can provide a systematic approach to examining the main effects of the process parameters—and the interactions between them—on the response variables. We have also observed that studies involving polymeric materials are scarce and, in the case of biocompatible materials, non-existent. Therefore, the aim of this paper is to investigate the effects of four process parameters: tool diameter, spindle speed, feed rate and step down on three response variables: forming axial force, surface roughness and final depth in the ISF process using two biocompatible thermoplastic materials: polycaprolactone (PCL) and ultra-high molecular weight polyethylene (UHMWPE). 

## 2. Materials and Methods

### 2.1. Geometry and Materials

**Figure 1:**
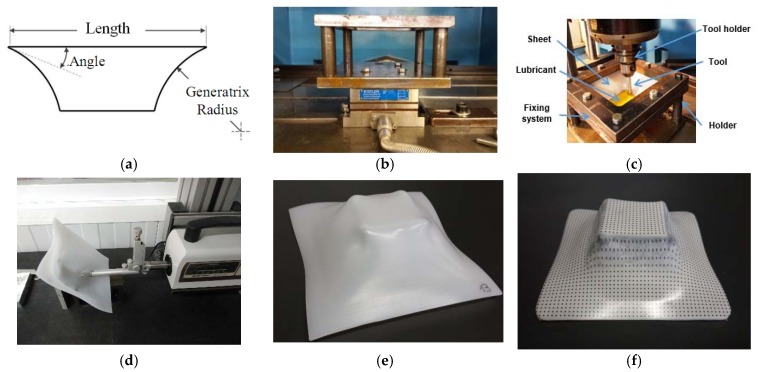
(**a**) Test geometry (**b**) Dynamometer with tooling (**c**) Experimental setup (**d**) Roughness measurement on ultra-high molecular weight polyethylene (UHWMPE) (**e**) UHMWPE part (**f**) polycaprolactone (PCL) part.

Tests were performed on 2 mm-thick sheets of the biocompatible polymers, UHMWPE and PCL. The test geometry in this experiment is a pyramidal frustum (Figure 1a), the features of which are:

- varying wall angle along the part’s depth.
- 105 mm in length.
- 45° for the initial wall angle
- 80 mm generatrix radius.

From a general point of view, both polymers present low density and high ductility but differ in their thermal properties, e.g., Vicat and melting temperature are lower for PCL (Table 1). The commercially available UHMWPE sintered sheets were initially around 10 mm thick and were sliced by a CNC saw machine and converted into 150 × 150 × 2 mm^3^ sheets. The PCL sheets were produced in our laboratory by compression molding. Around 55 g of PCL pellets (Sigma Aldrich, Saint Louis, MO, USA, ≈3 mm, average Mn = 80,000) were positioned into the cavity (150 × 150 × 2 mm^3^) of a stainless-steel cast which was previously warmed to a set temperature between 60 and 80 °C inside a heating hydraulic press. A low load was applied for a fixed time to guarantee the melt of the material. Subsequently, the load was increased, thus keeping the sheet in place for a few more minutes to complete the final compaction of the fused polymer. The pressure was then retired, and the cast cooled to room temperature by placing it in a cooling press.

The selection of these two materials is made according to two aspects, their mechanical behavior and their final application. They represent, in both cases, two confronted or extreme cases. From the point of view of mechanical behavior, PCL shows a decrease in strength after the yield point, although it is maintained in a stable value. Whereas for UHMWPE a strain hardening behavior is appreciated (in [16] where self-made PCL and UHMWPE sheets are compared) demonstrating that is a more rigid material. While under the point of view of the final biomedical application, they also respond to two different possible sectors where ISF technology can develop products. The characteristic of biodegradability is very important for PCL; this is why at present it is highly valued by doctors. While the basic properties of UHMWPE have been significant in the orthopedic sector for years.

### 2.2. Experimental Setup

The ISF tests were carried out using a Kondia^®^HS1000 3-axis milling machine (Kondia, Elgoibar, Spain). The details of the clamping system (Figure 1b) and the setup are described in detail in previous works [10]. The forming forces were measured by a table-type dynamometer Kistler^®^ 9257B (Kistler ibérica SL, Granollers, Spain); surface roughness was determined by means of a Mitutoyo Surftest SV-2000 profilometer (Sariki, Cerdanyola del Vallès, Spain) (Figure 1c) and the maximum depth was recorded by a direct reading off the Kondia milling machine (Kondia, Elgoibar, Spain).

### 2.3. Design of Experiments

Box-Behnken designs (BBD) [17] are three-level designs that allow second order response surfaces to be fitted efficiently. The design for the four factors consists of 27 experimental runs (Table 2) with 24 unique experimental settings (one replicate) plus three replicates at the central point. The explanatory variables came from the classical approach for metal ISF studies: thickness was kept constant for comparison and spindle speed was added because of its importance in polymer forming. The levels of the chosen variable were as follows:

- Dt: Tool diameter (6, 10, 14 mm)
- S: Spindle speed (Free*, 1000, 2000 rpm)
- F: Feed rate (1500, 2250, 3000 mm/min)
- Δz: Step down (0.2, 0.35, 0.5 mm).

* Rotation is considered to be free when any rotation of the tool is due solely to the friction between the sheet and the tool itself.

### 2.4. Analysis Procedure

**Figure 2:**
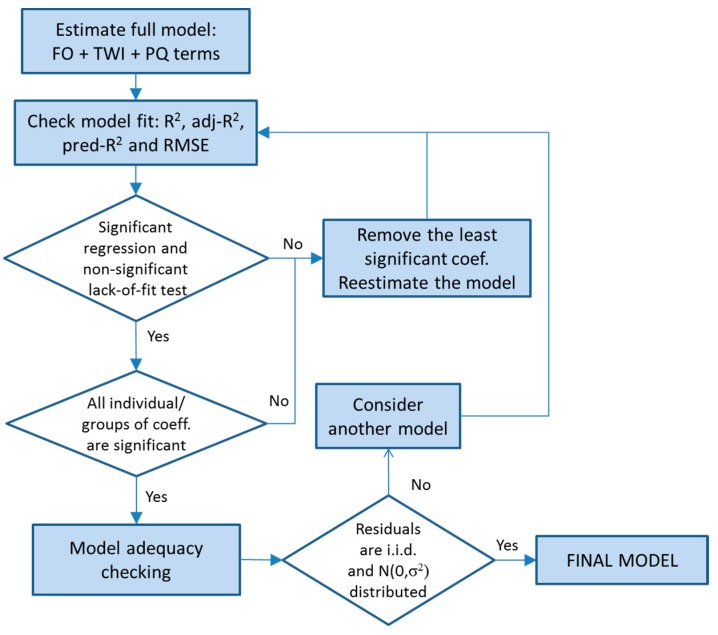
Model selection scheme.

The methodology used to estimate the response surface models is the one described by Myers [18]. The main steps are summarized in Figure 2 and are as follows:

- Estimation of the full model with first order, two-way interactions and pure quadratic terms.
- Sequentially removal of the non-significant terms based on the tests on individual regression and groups of coefficients. Each model was evaluated in terms of the fit statistics: R^2^, R^2^-adjusted, R^2^-predicted and RMSE. In addition, the test for significance of regression (*p*-value associated to Model in the ANOVA table) was observed: a *p*-value < α indicated that the regression was significant. The lack of fit test was also examined as an indicator of the tentative model satisfactorily describing the data when its *p*-value was high.
- Model adequacy checking: last squares regression assumptions.

The approach we followed was to retain, in the model, the smallest subset of explanatory variables providing a significant regression test and a non-significant lack-of-fit test as well as good fit statistics (high R^2^, adj-R^2^, pred-R^2^ and low RMSE) together with an appropriate model adequacy. The reason for using a subset of explanatory variables, rather than all of them, is that the estimates of the coefficients will have smaller variance and the predictions will be more precise. The Shapiro Wilk normality test (SWNT) was used to check the normality of the residuals.

Note that coded variables (−1, 0 and 1, indicating low, medium and high level, respectively) were used to compare the size of the coefficients and that the significance level in all cases is α = 0.05. The statistical analysis was conducted using R software [19].

## 3. Results and Discussion

The effects of varying various forming parameters on the three response variables under study (forming force, roughness and maximum depth) during an ISF process have been explored previously in a number of studies ranging from normal ISF to hot ISF, in experiments or simulations, and with regard to metals as well as polymers. We shall now go on to compare the results of these studies with the results we obtained for the two biocompatible materials in this work.

The factor levels (i.e., the process parameters) as well as response values for all 27 experimental runs are shown in Table 2. Note that the response value ΔRa has been introduced; we shall discuss the reasons for this in the following subsections, in which the statistical models proposed for each material are shown and briefly discussed.

### 3.1. Maximum Axial Force (Fz Max)

#### 3.1.1. PCL

The model that best fits Fz max for PCL is as follows:

(1) $$\hat{y}=304.83+92.12\cdot \mathrm{Dt}-31.01\cdot S-22.93\cdot \mathrm{Dt}\cdot S,$$

It is a very good model which describes 94% of the variability of Fz max and has a predicted R^2^ of 92%. The residuals of the model are homoscedastic, independent and identically normally distributed (SWNT *p*-value = 0.06).

Two of the first order factors, tool diameter (Dt) and spindle speed (S), are significant, as is the interaction between the two (Table 3) with Dt being the most influential because its coefficient is higher (three times higher than the coefficient of S). In general, the higher Dt (14 mm) produces higher Fz max values. It is clear that the portion of sheet to be formed using a greater tool diameter requires higher forces, as has been pointed out previously for metals (Al7075-O in Li et al. [6], Al3003-O in Bahoul et al. [7] and AISI304 in Centeno et al. [8]) as well as for polymers (PVC in Bagudanch et al. [9]). In contrast, an increase in spindle speed reduces the forces required. The mechanism able to explain this fact involves the change in the friction conditions, as the heat originating from the increase in friction decreases the various forming force values and, as a result, the mechanical behavior of the polymer material may change. This is also consistent with the work presented by Centeno et al. [8,20] and Baharudin et al. [21] for metal materials and, in the case of polymers, by Davarpanah et al. [11] for PLA and PVC, by Lozano-Sánchez et al. [16] for PCL and UHMWPE and by Bagudanch et al. [22] for PVC, PC, PP, UHMWPE and PCL.

The interpretation of the interaction effect (Dt·S) is as follows: a reduction in S (from 2000 rpm to 0 rpm) slightly increases Fz max when Dt = 6 mm, while the increment is more acute when Dt = 14 mm. As we have said, the increase in spindle speed increases the friction and temperature, causing lower force values, which agrees with results independently observed. Hence, it would be expected that, at higher tool diameters, the heat would concentrate even more in the forming region resulting in a temperature increase [22] which, in turn, would decrease the value of the required forming force; however, this does not occur according to the results we obtained. In the Dt·S interaction, it seems that the effect of the increased contact zone at higher tool diameters—which causes an increase in the forming force [4]—is a more dominant influence than the increase in temperature caused by the higher spindle speed.

#### 3.1.2. UHMWPE

The model that best explains Fz max on UHMWPE material is as follows:

(2) $$\hat{y}=626.33+187.88\cdot \mathrm{Dt}-98.82\cdot S-15.64\cdot F+6.40\cdot \Delta z-64.90\cdot \mathrm{Dt}\cdot S+46.34\cdot \mathrm{Dt}\cdot \Delta z+38.24\cdot S^{2}\;$$

This is a very good model able to explain 98% of the variability of Fz max and with a high capability of predicting new response values (pred R^2^ = 96%). The residuals of the retained model are i.i.d. distributed (SWNT *p*-value = 0.3303).

Like the PCL model, the UHMWPE model depends highly on Dt which is the most influential first order factor and also appears in the two significant two-way interactions. In general, high values of Dt result in higher values of Fz max. It is already known that an increase in tool diameter entails an increase in force. The second most influential factor is spindle speed, S, and it has a significant quadratic effect and interacts with Dt. The feed rate, F, is the least influential factor because of its lower coefficient; however, it is still significant. The effect of F can be appreciated in the (F, Dt) surface plot in Table 3; when F moves from 1500 to 3000 mm/min, Fz max is reduced on average by 15.64 N.

The interaction effects can be better understood from the surface plots. With regard to Dt·S, it can be appreciated how changing from S = 2000 rpm to S = 0 rpm increases Fmax in all cases, however this increment is more acute when Dt = 14 mm than it is when Dt = 6 mm.

The other significant interaction (Dt·Δz) has the opposite effect: on the one hand, when Dt = 6 mm, a reduction in Δz implies an increase in Fz max; on the other hand, when Dt = 14 mm, a reduction in Δz implies a reduction in Fz max. The reduction in step size decreases the portion of sheet being formed, which should also lead to a reduction in the force: the model shows this trend for high tool diameters. However, it is not the case for low tool diameters for which the forming force slightly increases.

### 3.2. Surface Roughness (Ra)

Roughness assessment needed to be conducted in a different way for each material, since the blank sheet was produced in different ways in each case. Furthermore, since it is known that sheet metal mark orientation can affect the results of roughness measures [23], it should be pointed out that the roughness values were obtained, in both cases, perpendicular to the step-down direction.

**Figure 3:**
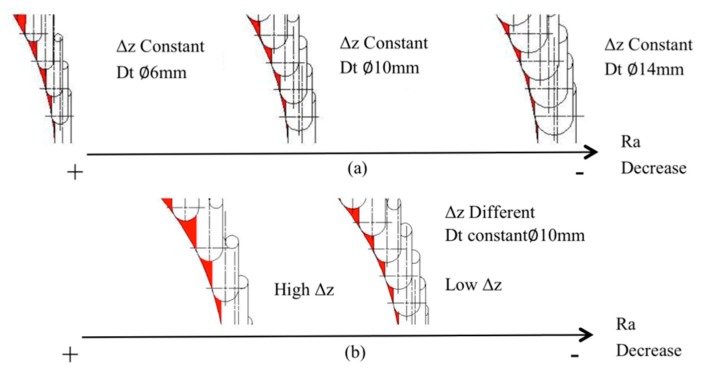
(**a**) Effect of different tool diameters, Dt, on surface roughness, Ra (**b**) Effect of different step down, Δz, on surface roughness, Ra.

Tool diameter and step down have been identified as parameters that influence surface roughness, Ra, [13,14] mainly in metals, whereas for PVC and PC, step down and spindle speed with some interactions were significant [23]. The manner in which the tool diameter and step down parameters improve or worsen the surface roughness can be seen in the diagrams in Figure 3, which shows how increased Dt reduces Ra, while increased Δz increases it.

#### 3.2.1. PCL

The model that best explains Ra for the PCL material includes all four factors of the experiment:

(3) $$\hat{y}=0.57+0.43\cdot \mathrm{Dt}+0.49\cdot S-0.17\cdot F+0.1\cdot \Delta z+0.42\cdot \mathrm{Dt}\cdot S-0.22\cdot F\cdot \Delta z+0.51\cdot S^{2}+0.16\cdot F^{2}+0.21\cdot {\Delta z}^{2}\;$$

The model explains 88% of the variability of the response Ra and has a good predicted R^2^ of 74%. Residuals are independent, homoscedastic and normally distributed (SWNT *p*-value = 0.3082). The model is complex because of the high numbers of terms included in it, but the surface plots shown in Table 4 can help in interpreting it.

In general, and in contrast to previous studies, a higher Dt is associated with a higher Ra (worse surface quality). However, this effect is more acute when S is at its high level (S = 2000 rpm), due to the friction and the heat that the surface is receiving [9]. Similarly, a higher Δz level is associated with a higher Ra, which is as expected, but again, this effect is more acute when the feed rate is low, F = 1500 mm/min, since at lower feed rates, contact time with the surface is higher, therefore there is more heat, which worsens surface quality. The effect of the quadratic terms S and F show a simple minimum pattern on the response surface. The stationary point of the response surface is Dt = 1.3, S = −1, F = 0.6, Δz = 0.1 in coded units or Dt = 15 mm, S = 0 rpm, F = 2688 mm/min, Δz = 0.36 mm. This stationary point represents a minimum for the Ra value.

#### 3.2.2. UHMWPE

The parameter of roughness for UHMWPE analyzed here will be the difference between the roughness before and after processing (ΔRa = Rabefore processing–Raafter processing). In contrast to PCL in which the sheets proceed direct from a mold, in the case of UHMWPE, the sheets were sliced in layers from a thicker molded sheet, which meant the surface already began with a texture whose starting roughness was high, at around 1.05 μm on average. With one exception, the ΔRa value is always positive, which means that the processing operation improves the roughness of the material. The roughness is reduced on average by 0.464 μm with 95% CI = [0.36, 0.57].

The model selected to explain the differences in roughness in UHMWPE is as follows:

(4) $$\hat{y}=0.46+0.08\cdot \mathrm{Dt}-0.03\cdot S-0.03\cdot F-0.36\cdot \mathrm{Dt}\cdot S\;$$

The model, however, explains only 21% of the variability of the response variable and has a bad predicted R^2^. It was decided to keep F in the model to achieve a non-significant lack-of-fit test. The general regression test is significant. Residuals are normally distributed (SWNT *p*-value = 0.87).

The most important factor explaining the differences in roughness in UHMWPE is the interaction between Dt and S which has a negative coefficient. The surface plots shown in Table 4 can help interpret the model. They show a saddle point (the stationary point) near the center of the plot. From the center point, increasing or decreasing Dt and S at the same time produces a reduction in ΔRa, while increasing one factor and decreasing the other leads to an increase in ΔRa. In other words, Ra after processing is slightly reduced when Dt and S are both at their most positive or most negative values (low ΔRa values), whereas Ra is highly reduced when Dt and S are at opposite levels (very low ΔRa values).

### 3.3. Maximum Achieved Depth (Zmax)

The response variable, maximum achieved depth, Zmax, both on UHMWPE and PCL, cannot be analyzed using a response surface model because the data shows a highly right-skewed distribution which is truncated at different depths (42.7 mm for a step down of 0.35 mm or 43.0 mm in the other cases) depending on the experimental settings. For example, for UHMWPE, 20 out of 27 experiments reached the maximum specified depth (Zmax = 100%), five were in the interval [90,100) and two in the interval [80,90). All tested models have a significant lack of fit test and show a dependence on the residuals vs. fitted values, mainly when Zmax = 100%.

The reason for analyzing the Zmax response is to find the point, z, at which the material breaks and to find which factors explain the breaking depth (Zmax). Generally, this can be modeled using survival (or reliability) analysis which aims to analyze the relationship of time to an event. In this context, the event is defined as the breaking (or failure) of the polymer and time is represented by the depth, z.

The experimental data shows many censored observations, that is, experiments in which the material did not break. Each experiment is an observation of the type (zi,ci) where zi is Zmax and ci = 1 if there has been a failure, and ci = 0 (censored) if otherwise. These ci values are shown in parentheses in Table 2 alongside the Zmax values.

The aim of this section is to explain the probability of the material surviving at depth z, that is, the survival, or reliability, function: S(z) = 1 − F(z) = P(Z ≥ z)–being careful not to confuse S(z) with the factor S = Spindle-speed. Another important function in survival analysis is the hazard function, λ(z). It assesses the instantaneous risk of demise at next mm, conditional on survival to that depth. In other words, it is the expected number of events that will occur in the next mm given that there has not been an event to that depth (z).

In this analysis, the Kaplan Meyer method (a non-parametric approach) is used to estimate S(z). Moreover, the Mantel-Haenszel test (log-rank test) is used to check for differences between survival curves (factor levels).

It is finished by fitting a Cox proportional hazard regression model (semi parametric approach) to determine whether the model including the significant factors is significant itself and to look for significant interactions. It is used with the likelihood ratio, Wald and logrank tests.

Note that the number of experiments at each level is not balanced. For example, there are six experiments at S = −1, 15 at S = 0 and 6 at S = 1. This is because data was not collected to carry out a survival analysis, but rather a BBD of experiments.

#### 3.3.1. PCL

**Figure 4:**
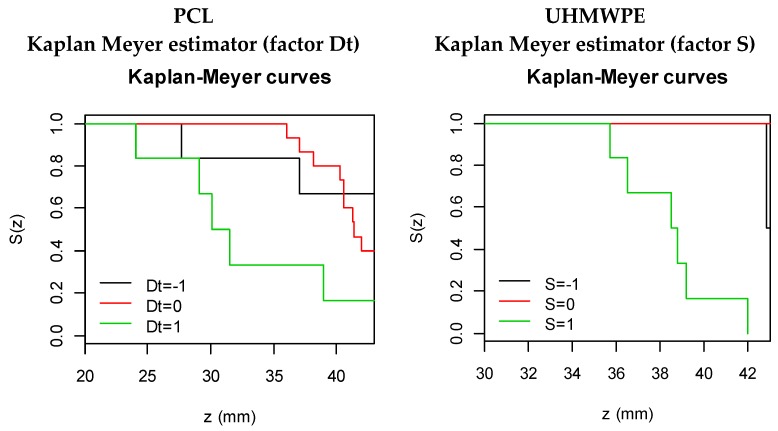
Significant survival curves according to factor levels on PCL and UHMWPE.

Kaplan Meyer curves (Figure 4) can be interpreted as follows: at z = 20 mm, all observations/experiments are without event and the survival (S(z)) is 1, or, equivalently, 100%. The solid lines, colored according to factor levels, show the events with vertical drops (at each z). Colored curves showing different patterns indicate that there are differences in survival between factor levels, such as is the case of factor Dt (Mantel-Haenszel test *p*-value = 0.033): it is the only significant factor in PCL survival.

Note that the survival curves are not balanced: there are more experiments on Dt = 10 mm than on Dt = 6 or 14 mm (15 vs. 6). However, it is noticeable that survival is lower for Dt = 14 mm.

The Cox proportional hazard regression model has a unique significant factor: Dt. The coefficient of S (b = 0.95) is interpretable in its exponential form (e^0.95^ = 2.59) as the multiplicative effects of the hazard. That is, increasing Dt one level (e.g., from −1 to 0) increases the danger of breaking by, on average, a factor of 2.59. The overall tests of significance *p*-value (likelihood ratio, Wald and logrank) are significant, indicating that the model is appropriate.

Nevertheless, when S is included in the Cox model together with Dt, the individual regression coefficient of S has a *p*-value of 0.07 and the overall model is significant. In this case, the coefficient associated with S is b = −0.70 meaning that increasing S by one level reduces the danger of breaking by e^−0.70^ = 0.50. Note, however, that the interaction between Dt and S is not significant. More experiments should be carried out in order to confirm the significance of S in the survival function.

#### 3.3.2. UHMWPE

Kaplan Meyer curves for Zmax on UHMWPE show significant differences only across S levels (Mantel-Haenszel test *p*-value < 0.001). From Figure 4, it can be seen that S(z) is lower when S = 2000 rpm. No breaks were observed for S = 1000.

The Cox proportional hazard regression model has S as a unique significant factor. Its coefficient is $\hat{\beta }=3.38$ meaning that increasing S one level increases, on average, the danger of breaking by e^3.38^ = 29.49.

**Figure 5:**
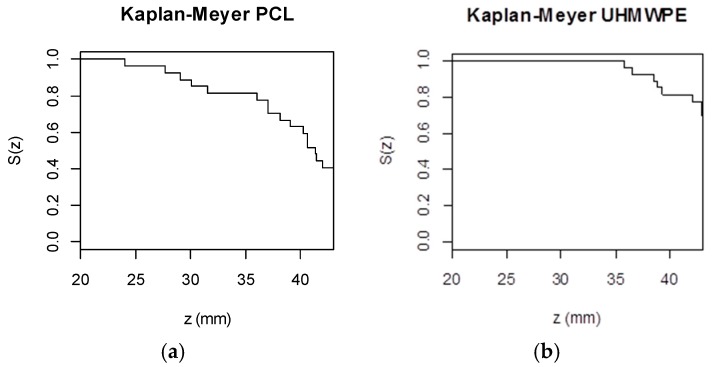
Kaplan-Meyer curves without separating by factor levels for (**a**) PCL and (**b**) UHMWPE.

The Kaplan-Meyer curves are shown globally without being separated by factor levels in Figure 5. In the graphics comparing PCL versus UHMWPE, it can be observed that PCL sheets break earlier (z around 25) and in greater quantity than UHMWPE sheets in which the first breakage appears at around z = 35.

## 4. Conclusions

In this work, SPIF experimental tests using two different polymers biocompatible have been carried out following a Box-Behnken design for four factors and a survival analysis. The maximum forming force, surface roughness and Zmax response achieved in the experiments has been statistically analyzed and empirical models for each material have been obtained. Using the proposed models, it is possible to control the Fz max, Ra and Zmax.

Among the process parameters analyzed and according to the data summarized in Table 5, spindle speed and tool diameter have been found to be the most influential parameters in terms of maximum forming force variation for both materials. From the roughness analysis, one can observe again the importance of spindle speed and tool diameter on these biocompatible polymers, as both involve an increase in temperature due to either the friction at higher spindle speeds or the increase in surface contact and contact time between tool and sheet, both of which worsen the surface because the materials flow, degrade and lose their properties.

Finally, the response Zmax, on UHMWPE and PCL, cannot be analyzed using a response surface model because data is highly right-skewed and truncated at different depths. However, taking into account the objective of the paper, the data can be analyzed using a novel method in this field: survival analysis. The results have shown that the most important factor is spindle speed for UHMWPE and tool diameter tool for PCL.

## Figures and Tables

**Table 1:** Mechanical properties.

Material	Vicat Softening Temperature (°C)	Tensile Strength (MPa)	Elastic Modulus (MPa)
PCL	44.3	15.2	375
UHMWPE	80	19	700

**Table 2:** Design of experiments and results.

ID	Tool Diameter, Dt (mm)	Spindle Speed, S (rpm)	Feed Rate, F (mm/min)	Step Down, Δz (mm)	PCL			UHMWPE		
					Fz Max (N)	Ra (μm)	Zmax * (mm)	Fz max (N)	ΔRa (μm)	Zmax * (mm)
1	6	Free	2250	0.35	208.72	0.498	27.7 (0)	485.33	0.437	42.7 (1)
2	14	Free	2250	0.35	439.14	0.627	29.1 (0)	1027.50	0.750	42.7 (1)
3	6	2000	2250	0.35	190.95	0.41	42.7 (1)	414.58	0.916	42.0 (0)
4	14	2000	2250	0.35	329.64	2.23	43.0 (1)	697.15	−0.194	35.7 (0)
5	10	1000	1500	0.20	314.38	0.608	41.4 (0)	635.68	0.511	43.0 (1)
6	10	1000	3000	0.20	309.16	0.622	43.0 (1)	596.08	0.242	43.0 (1)
7	10	1000	1500	0.50	293.05	1.393	42.0 (0)	636.66	0.391	43.0 (1)
8	10	1000	3000	0.50	291.63	0.509	43.0 (1)	591.00	0.324	43.0 (1)
9	10	1000	2250	0.35	325.57	0.585	38.2 (0)	643.44	0.477	42.7 (1)
10	6	1000	2250	0.20	214.14	0.453	43.0 (1)	491.68	0.508	43.0 (1)
11	14	1000	2250	0.20	425.94	1.114	39.0 (0)	765.37	0.739	43.0 (1)
12	6	1000	2250	0.50	197.63	0.484	37.0 (0)	399.84	0.373	43.0 (1)
13	14	1000	2250	0.50	390.87	1.549	24.0 (0)	858.88	0.635	43.0 (1)
14	10	Free	1500	0.35	320.78	1.023	40.6 (0)	818.05	0.332	42.7 (1)
15	10	2000	1500	0.35	275.32	1.880	41.3 (0)	581.63	0.230	38.5 (0)
16	10	Free	3000	0.35	343.10	0.716	40.3 (0)	747.58	0.198	42.7 (1)
17	10	2000	3000	0.35	282.30	1.735	40.6 (0)	558.92	0.420	39.2 (0)
18	10	1000	2250	0.35	296.22	0.464	42.7 (1)	595.00	0.524	42.7 (1)
19	6	1000	1500	0.35	227.42	0.527	42.7 (1)	486.95	0.354	42.7 (1)
20	14	1000	1500	0.35	418.57	1.385	30.1 (0)	802.43	0.863	42.7 (1)
21	6	1000	3000	0.35	240.99	0.330	42.7 (1)	449.30	0.231	42.7 (1)
22	14	1000	3000	0.35	381.09	0.902	31.5 (0)	830.85	0.956	42.7 (1)
23	10	Free	2250	0.20	330.25	0.859	36.0 (0)	727.72	0.115	42.8 (0)
24	10	2000	2250	0.20	281.23	1.775	37.0 (0)	554.77	0.549	38.8 (0)
25	10	Free	2250	0.50	355.89	0.579	43.0 (1)	774.19	0.801	43.0 (1)
26	10	2000	2250	0.50	266.34	2.102	43.0 (1)	587.48	0.407	36.5 (0)
27	10	1000	2250	0.35	280.30	0.598	42.7 (1)	611.80	0.440	42.7 (1)

Note: * In parentheses: 1 = maximum depth accomplished, 0 = sheet fracture.

**Table 3:** Maximum force (Fz max) model results.

**PCL**						**UHWMPE**					
**Parameter estimates**						**Parameter estimates**					
	Coefficient			*p*-value			Coefficient			Pr (>|t|)	
(Intercept)	304.84			<0.001		(Intercept)	626.33			<0.001	
Dt	92.12			<0.001		Dt	187.88			<0.001	
S	−31.01			<0.001		S	−98.82			<0.001	
Dt·S	−22.93			0.009		F	−15.64			0.033	
						Δz	6.40			0.359	
						Dt·S	−64.90			<0.001	
						Dt·Δz	46.34			<0.001	
						S^2^	38.24			<0.001	
**Analysis of variance**						**Analysis of variance**					
	Df	Sum Sq	Mean Sq	F value	*p*-value		Df	Sum Sq	Mean Sq	F value	*p*-value
Model	3	115,468	38,489	149.76	<0.001	Model	7	579,363	82,766	148.86	<0.001
Residuals	23	5918	257			Residuals	19	10,570	556		
Lack of fit	5	1185	237	0.90	0.502	Lack of fit	17	9360	551	0.91	0.644
Pure Error	18	4733	263			Pure Error	2	1210	605		
**Summary of fit**						**Summary of fit**					
R^2^	0.95		RMSE	16.04		R^2^	0.98		RMSE	23.59	
Adj, R^2^	0.94		Pred. R^2^	0.93		Adj, R^2^	0.98		Pred. R^2^	0.96	
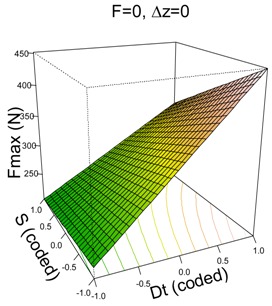						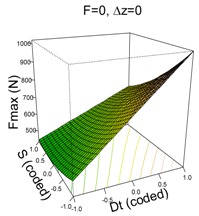					
						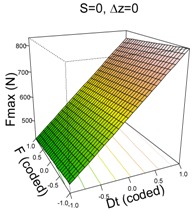					
						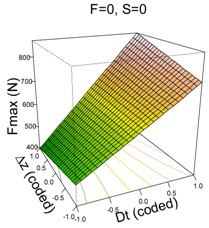					

**Table 4:** Surface Roughness (Ra) model results.

**PCL**						**UHWMPE**					
**Parameter estimates**						**Parameter estimates**					
	Coefficient			*p*-value			Coefficient			*p*-value	
(Intercept)	0.57			<0.001		(Intercept)	0.46			<0.001	
Dt	0.43			<0.001		Dt	0.08			0.259	
S	0.49			<0.001		S	−0.03			0.707	
F	−0.17			0.010		F	−0.03			0.703	
Δz	0.10			0.105		Dt·S	−0.36			0.006	
Dt·S	0.42			0.001							
F· Δz	−0.22			0.038							
S^2^	0.51			<0.001							
F^2^	0.16			0.070							
Δz ^2^	0.21			0.020							
**Analysis of variance**						**Analysis of variance**					
	Df	Sum Sq	Mean Sq	F value	*p*-value		Df	Sum Sq	Mean Sq	F value	*p*-value
Model	9	7.97	0.89	22.24	<0.001	Model	4	0.59	0.15	2.77	<0.001
Residuals	17	0.68	0.04			Residuals	22	1.18	0.05		
Lack of fit	15	0.67	0.04	8.14	0.115	Lack of fit	14	0.91	0.06	1.89	0.184
Pure Error	2	0.01	0.01			Pure Error	8	0.27	0.03		
**Summary of fit**						**Summary of fit**					
R^2^	0.92		RMSE			0.20	R^2^	0.33		RMSE	0.23
Adj, R^2^	0.88		Pred. R^2^			0.74	Adj, R^2^	0.21		Pred. R^2^	−0.33
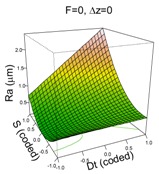						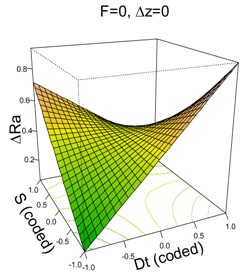					
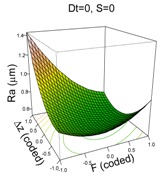											
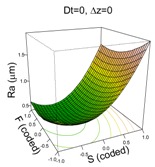											

**Table 5:** Summary of the coefficients of the selected models (bold indicates significance (α = 0.05)).

	Intercept	Dt	S	F	Δz	Dt·S	Dt·Δz	F·Δz	S^2^	F2	Δz^2^
**Fz max**											
PCL	304.84	92.12	−31.01			−22.93					
UHMWPE	626.33	187.88	−98.82	−15.64	6.40	−64.90	+46.34		38.24		
**Ra**											
PCL	0.57	0.43	0.49	−0.17	0.1	0.42		−0.22	0.51	0.16	0.21
UHMWPE (ΔRa)	0.46	0.08	−0.03	−0.03		−0.36					
**Z max (Survival analysis)**											
PCL		**×**									
UHMWPE			**×**								
